# Case Report: Clinical response to TKI therapy in T-cell acute lymphoblastic leukemia with rare *ABL1* rearrangement

**DOI:** 10.3389/fonc.2026.1928646

**Published:** 2026-09-10

**Authors:** Mingyan Zhong, Wenting He, Lingji Zeng, Jinfang Zhang

**Affiliations:** 1 Department of Paediatric Hematology, Guangdong Provincial People’s Hospital, Guangdong Academy of Medical Sciences, Southern Medical University, Guangzhou, Guangdong, China; 2 Department of Physical Examination and Health Management, Guangdong Provincial People’s Hospital, Guangdong Academy of Medical Sciences, Southern Medical University, Guangzhou, Guangdong, China; 3 Department of Hematology, Guangdong Provincial People’s Hospital, Guangdong Academy of Medical Sciences, Southern Medical University, Guangzhou, Guangdong, China

**Keywords:** *ABL1* rearrangement, ALL, T-cell acute lymphoblastic leukemia, TKIs, *ZBTB16::ABL1*

## Abstract

**Background:**

T-cell acute lymphoblastic leukemia (T-ALL) with *ABL1* rearrangement is an extremely rare disease, and its clinical significance has not been precisely defined. We report the clinical characteristics of a pediatric patient with T-cell acute lymphoblastic leukemia harboring *ABL1* rearrangement and the treatment response to tyrosine kinase inhibitor (TKI) therapy, providing a reference for clinical management with this subtype.

**Case reports:**

A 3-year-old boy with T-ALL and *ZBTB16::ABL1* fusion (RNA-seq) showed poor response to induction chemotherapy, failing remission at D33. Dasatinib (75 mg/m^2^/day) was added concurrently with CAM chemotherapy. At D90, morphological CR and flow-MRD negativity were achieved, though qPCR remained positive at 0.08%. Isolated CNS relapse occurred during Block III consolidation. Subsequent management included autologous CD7 CAR T-cell therapy and allogeneic HSCT; post-transplant evaluation confirmed sustained leukemia remission. However, the patient developed HHV-6 encephalitis and adenovirus hepatitis with hepatic failure; life-sustaining support was withdrawn at the family’s request. The patient was discharged and lost to follow-up.

**Conclusion:**

This paper presents the second reported case to date of TKI treatment for T-cell acute lymphoblastic leukemia harboring the *ZBTB16::ABL1* fusion. Following a review of the relevant literature on *ABL1*-rearranged T-ALL, we conclude that the accumulation of additional cases is essential to better characterize this rare molecular subtype and to guide evidence-based treatment decisions.

## Introduction

T-cell acute lymphoblastic leukemia (T-ALL) is an aggressive hematologic malignancy caused by the abnormal proliferation of T-cell precursors. Although intensive chemotherapy and hematopoietic stem cell transplantation have significantly improved outcomes, the treatment of relapsed/refractory T-ALL remains challenging, especially for patients with specific molecular abnormalities (1). Recent advances in molecular diagnostics have enabled the identification of rare driver gene fusions offering new directions for precision classification and targeted therapy in T-ALL (2).

*ABL1* rearrangements are extremely rare in T-ALL, the corresponding partner genes remain incompletely characterized. To date, *NUP214::ABL1* is the most frequently identified *ABL1* fusion in T-ALL, while *BCR::ABL1* is considerably less common in this subtype. Other fusion variants, such as *ETV6::ABL1* or *EML1::ABL1*, have also been documented but occur at lower frequencies (3). Furthermore, the correlation between *ABL1* rearrangement and clinical therapeutic response to Tyrosine kinase inhibitors (TKIs) remains unclear.

The *ZBTB16::ABL1* fusion gene is an extremely rare molecular abnormality in T-ALL, and its clinical significance is still unclear. This fusion results from the rearrangement of the zinc-finger transcription factor gene *ZBTB16* (also known as *PLZF)* with the tyrosine kinase gene *ABL1*, leading to constitutive activation of the ABL1 kinase domain (4). Tyrosine kinase inhibitors such as dasatinib, which target abnormal *ABL1* kinase activity, have shown efficacy in *BCR::ABL1* positive leukemia (5). However, the therapeutic impact of TKIs on *ZBTB16::ABL1* remains unclear. To date, no studies have comprehensively characterized the clinical features and drug response profiles associated with this fusion gene based on individual cases or case series. Its molecular mechanism, clinical prognosis, and optimal TKI treatment regimen still require further investigation. In conclusion, T-ALL with *ABL1* rearrangement still necessitates more in-depth research.

## Case presentation

A 3-year-old boy was admitted to our hospital because of a fever for 2 days and leukocytosis for 1 day. Initial laboratory tests revealed the following: WBC count 134.22 × 10^9^/L, hemoglobin 118 g/L, platelet count 64 × 10⁹/L, D-dimer 4130 ng/mL, and LDH 346 U/L. Physical examination revealed generalized lymphadenopathy and hepatosplenomegaly.

**Figure 1:**
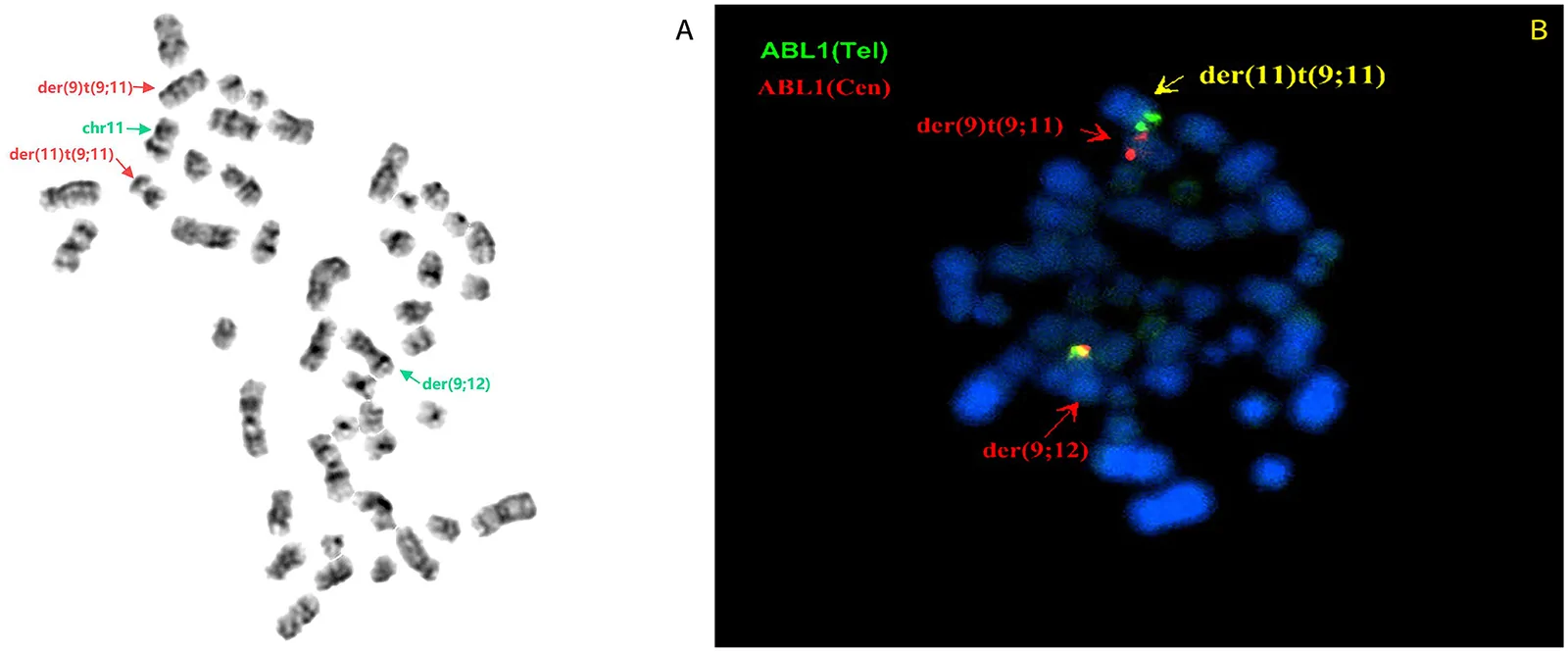
Chromosomes and genetic testing results. **(A)** Partial G-banding karyotype showing the aberrations (×1000): 45,XY,t(9;11)(q34;q23),der(9;12)(q10;q10),del(14)(q24)[15]/46,XY[5]. **(B)** Metaphase FISH on chromosomes 9 with an ABL1 break-apart probe (×640). The probe located centromeric to the ABL1 gene labelled in red and telomeric to the ABL1 gene labelled in green.

**Figure 2:**
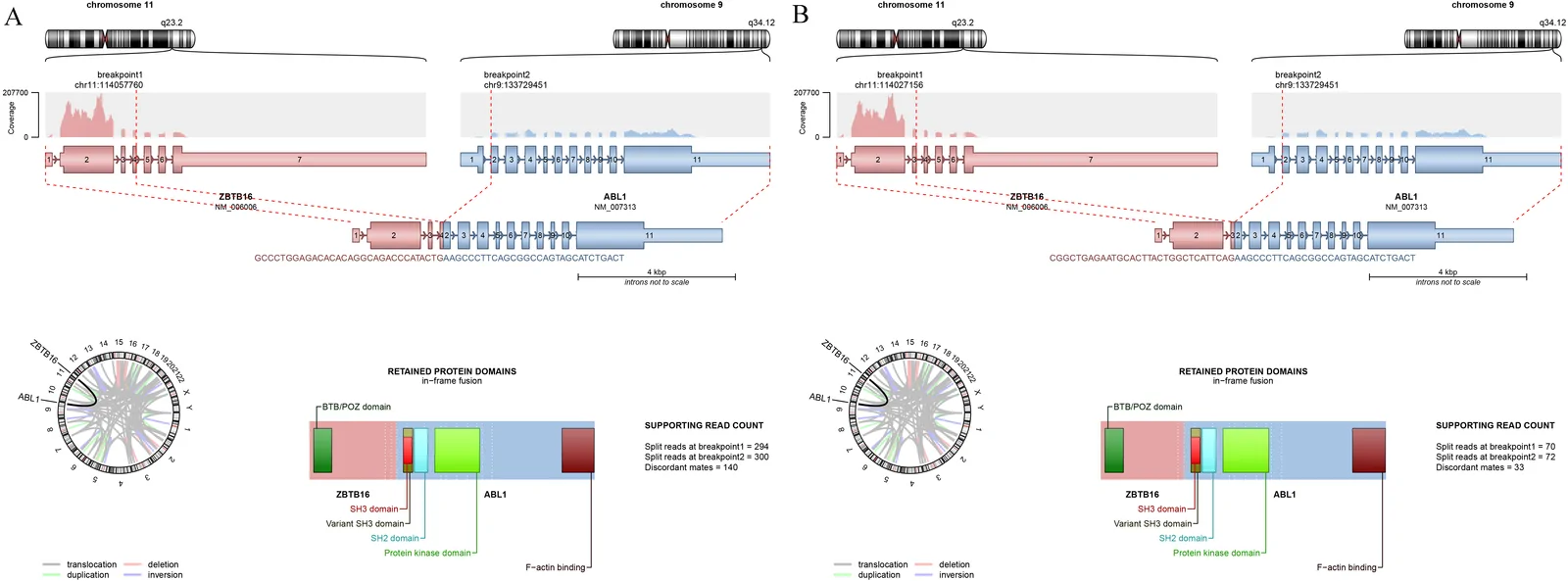
*ZBTB16::ABL1* fusion transcripts. **(A)** The sequencing results of targeted RNAseq fusion genes suggest that one of the *ZBTB16::ABL1* fusion genes is downstream of exon 4 of the *ZBTB16* gene (chr11, NM_006006, with a total of 7 exons) connected to the *ABL1* gene (chr9, NM_005157.6). Upstream of exon 2 (a total of 11 exons), a fusion gene within a frame (without frame shift) is formed; **(B)** Another *ZBTB16::ABL1* fusion gene is that the downstream of exon 3 of the *ZBTB16* gene (chr11, NM_006006, a total of 7 exons) is connected to the upstream of exon 2 of the *ABL1* gene (chr9, NM_005157.6, a total of 11 exons). Form a fusion gene within a box (without box shift).

A bone marrow aspirate was performed after admission. The bone marrow showed markedly hypercellular proliferation with a G/E ratio of 11:1, and 76.5% of nucleated cells were blasts. Bone marrow flow cytometry was consistent with T-cell lymphoblastic leukemia/lymphoma. Aberrant cells comprised 67.52% of the total nucleated cells and expressed CD71 (dim), CD117 (partial/dim), CD38 (dim), CD5, surface CD3, CD8, CD7, CD56, cytoplasmic CD3, and TCR γ/δ. These cells were negative for CD123, CD34, HLA-DR, CD15, CD11b, CD14, CD4, CD64, CD13, CD22, CD33, CD19, CD10, CD20, CD27, CD11c, CD61, CD41, TdT, CD1a, TCR α/β, CD9, cytoplasmic CD79a, CD2, and MPO. Cytogenetic analysis revealed a complex karyotype: 45, XY, t(9;11)(q34;q23), der(9;12)(q10;q10), del(14)(q24)[15]/46,XY[5] (**Figure 1A**). Fluorescence *in situ* hybridization (FISH) showed *ABL1* rearrangement in 75% of nuclei (**Figure 1B**), other common fusions were negative, including *BCR::ABL1, EPOR, CRLF2, PDGFRB, JAK2, TEL::AML1 (ETV6::RUNX1), E2A (TCF3)*, and *MLL (KMT2A)*. To identify the specific fusion partner, RNA sequencing was performed and identified two in-frame *ZBTB16::ABL1* fusion transcripts. The predominant isoform resulted from the fusion of *ZBTB16* exon 4 (located on chromosome 11, NM_006008, breakpoint chr11:114057760) with *ABL1* exon 2 (located on chromosome 9, NM_007313, breakpoint chr9:133729451), with 294 split reads supporting the 5’ junction and 300 split reads supporting the 3’ junction. A minor isoform resulted from the fusion of *ZBTB16* exon 3 (breakpoint chr11:114027156) with *ABL1* exon 2 (breakpoint chr9:133729451), with 70 split reads at the 5’ junction and 72 split reads at the 3’ junction. Both transcripts are predicted to encode in-frame fusion proteins retaining the BTB/POZ domain of *ZBTB16* and the SH3 domain, variant SH3 domain, protein kinase domain, and F-actin-binding domain of *ABL1*. The fusion was confirmed by RT-PCR followed by Sanger sequencing (**Figure 2**). Targeted high-throughput sequencing of a 130-gene panel associated with hematological malignancies was performed, and no Tier I pathogenic variants were identified per the AMP/ASCO/CAP classification (6).

The patient was diagnosed with acute lymphoblastic leukemia (T-cell lineage, *ZBTB16::ABL1* positive) and was treated according to the South China Children’s Leukemia Group–ALL-2016 protocol (SCCLG-ALL-2016). This trial is registered with the Chinese Clinical Trial Registry (Chi-CTR; number ChiCTR2000030357) (**Supplementary material**). Treatment was initiated with a prednisone sensitivity test, which revealed a poor response. Induction therapy was then commenced using the VDLD + CTX regimen. During induction, five lumbar punctures with intrathecal chemotherapy were performed; cerebrospinal fluid analysis showed no abnormalities. On day 15 (D15), bone marrow aspiration indicated active disease with 64.0% lymphoblasts. Minimal residual disease assessed by flow cytometry (FCM-MRD) detected approximately 56.66% leukemic cells expressing CD7, CD5, CD3, and partial CD56. Then on day 33 (D33), repeat bone marrow smear showed continued hypercellular marrow with 13.0% lymphoblasts. FCM-MRD revealed approximately 8.18% residual leukemic cells with the same immunophenotypic profile (CD7+, CD5+, CD3+, partial CD56+). Thus, the patient failed to achieve remission at D33 and was clinically stratified into the high-risk group.

Subsequently, dasatinib (75 mg/m^2^/d, administered orally once daily) targeted therapy was initiated alongside CAM chemotherapy. On day 90, bone marrow smear revealed CR and FCM-MRD was negative, while quantitative PCR for the *ZBTB16::ABL1* fusion transcript in the bone marrow showed 0.08%. The patient proceeded to consolidation therapy with Block regimens. During Block III, cerebrospinal fluid (CSF) examination revealed 2.11% (1,445 cells) of immature T lymphocytes in the CSF-MRD specimen, expressing CD3, CD5, and CD7. CSF cytology showed >20 nucleated cells per high-power field (HPF), including 3% blasts. Meanwhile, bone marrow smear again confirmed CR, and no MRD-positive cells were detected by FCM. These findings were consistent with a diagnosis of CNS relapse of T-ALL (isolated, without clinical neurological symptoms).

**Figure 3:**
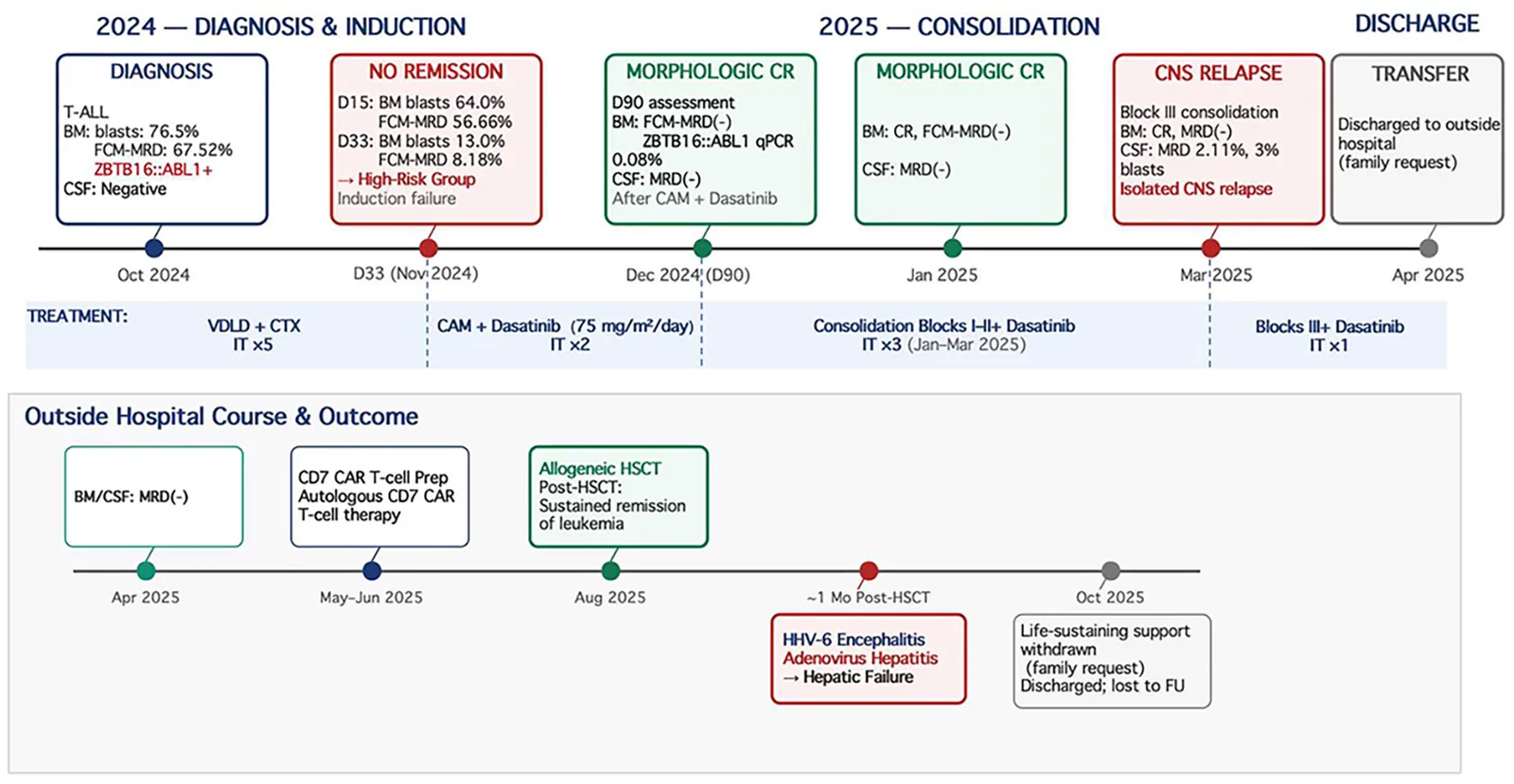
Illustration of the treatment process. The diagram summarizes the disease course, therapy, and outcomes. Initial BM blasts were 76.5% (FCM-MRD 67.52%). Induction with VDLD + CTX failed (Day 33 BM blasts 13.0%, FCM-MRD 8.18%), leading to high-risk stratification. CR was achieved at Day 90 after CAM + dasatinib (BM/CSF MRD-negative; ZBTB16::ABL1 qPCR 0.08%). Consolidation with dasatinib maintained remission until an isolated CNS relapse occurred during Block III. After transfer, the patient received autologous CD7 CAR T-cell therapy followed by allogeneic HSCT. At ~1 month post-HSCT, HHV-6 encephalitis and adenovirus hepatitis caused hepatic failure; supportive care was withdrawn in October 2025 per family request, and the patient was lost to follow-up. BM, bone marrow; CNS, central nervous system; CR, complete remission; CSF, cerebrospinal fluid; FCM-MRD, flow cytometry-based minimal residual disease; HSCT, hematopoietic stem cell transplantation; IT, intrathecal therapy; qPCR, quantitative polymerase chain reaction; FU, follow-up.

After completing consolidation therapy, the patient was transferred to an outside hospital at the family’s request. Reevaluation at that institution demonstrated MRD-negative remission in both bone marrow and CSF. The patient subsequently received CD7 CAR T-cell therapy and allogeneic HSCT. Although post-transplant evaluation confirmed sustained remission, HHV-6 encephalitis and adenovirus hepatitis developed approximately one month after HSCT, progressing to hepatic failure. Life support was withdrawn at the family’s request, and the patient was discharged and subsequently lost to follow-up (**Figure 3**).

## Discussion

The *ABL1* rearrangements are rare but pathogenic molecular abnormalities in T-ALL, and the clinical implications and optimal therapeutic strategies are still under investigation. Distinct from Ph+ or Ph-like B-ALL, *ABL1* rearrangement may confer unique biological characteristics and clinical outcomes in T-ALL (7). This study combines existing literature with clinical observations to describe the clinical course of a T-ALL case harboring the *ZBTB16::ABL1* fusion and to contextualize this finding within the limited available evidence.

Among T-lineage acute lymphoblastic leukemia (T-ALL) cases with *ABL1* rearrangements, *NUP214::ABL1* is the most frequently identified fusion (7–9), whereas *BCR::ABL1* is considerably less common in this subtype (10–13). Other fusion variants, including *ETV6::ABL1* and *EML1::ABL1*, have also been reported in sporadic cases (14, 15). According to existing limited reports, *BCR::ABL1*-positive T-ALL is associated with poor prognosis. However, several reports have documented that TKIs combined with chemotherapy achieves favorable therapeutic outcomes in patients with *BCR::ABL1* positive T-ALL (10). *ZBTB16::ABL1* fusions is extremely rarely reported in existing literature, and their prognostic significance remains unclear (4, 16). We herein briefly summarize the currently reported *ABL1* rearrangement subtypes in T-ALL (**Table 1**). Given the rarity of these fusions, definitive conclusions regarding their prognostic impact cannot be drawn, and further investigation is warranted.

**Table 1:** ABL1 rearrangement subtypes in T-ALL.

Fusion gene	Karyotype	Number of cases	TKIs	References
*NUP214::ABL1*	Episomes/hsr	63	Dasatinib/bosutinib	Hagemeijer A et al., 2010 (7)
		4	None	Patra N et al., 2021 (8)
		1	Dasatinib	Zhu L et al., 2025 (9)
*BCR::ABL1*	t(9;22)(q34;q11.2)	31	Imatinib Dasatinib	Xuewei Li et al., 2021.
	t(9;22) (q34;q11.2)	1	Imatinib Dasatinib nilotinib	Kohla S et al., 2021 (11).
	t(9;22)(q34;q11.2)	1	dasatinib	Dong Q et al., 2024 (12).
	t(9;22)(q34.12;q11.23)	1	Nilotinib Ponatinib	Shah S et al., 2024 (13).
*ETV6::ABL1*	47,XXYc,del(6)(q15q23),der(9)ins(9;12)(q34;p13p13)inv(9)(q34q34) Rarrangement between 9q34 and 12p13	1	None	van Limbergen et al., 2001 (14)
*EML1::ABL1*	t(9;14)(q34;q32)	1	Imatinib	de Keersmaecker et al., 2005 (15)
*ZBTB16::ABL1*	t(9;11)(q34;q23)	2	None	Chen B et al., 2018 (4)
		1	Imatinib	Z. Krenova et al., 2022 (16)

The *ZBTB16::ABL1* fusion leads to constitutive activation of the ABL1 kinase domain, driving leukemogenesis via pathways such as *JAK/STAT, PI3K/AKT*, and *RAS/MAPK* (4). Unlike *BCR::ABL1, ZBTB16::ABL1* may exhibit unique biological behaviors due to the transcriptional regulatory properties of *ZBTB16* (17). The *ZBTB16* gene, also known as *PLZF* (promyelocytic leukemia zinc finger), belongs to the Krueppel C2H2 type zinc finger protein family. The protein product encoded is a zinc finger transcription factor. It contains one BTB domain and nine Kruppel-type zinc finger domains at the C-terminus. The *ZBTB16* protein is located in the cell nucleus, participates in the progression of the cell cycle, and can interact with histone deacetylases. There have been reports of cleavage rearrangement of the *ZBTB16* gene (*ZBTB16::RARA*) in acute promyelocytic leukemia (18). *ABL1* is a proto-oncogene and a common non-receptor tyrosine kinase expressed by cells. *ABL1* is located in the cytoplasm and nucleus and can be activated by growth factor receptors, cell kinases or DNA damage (19). The *ZBTB16::ABL1* fusion gene detected in this case likely results from t (9; 11)(q34; q23.2). It is caused by translocation events and is a relatively rare fusion. The fused N-terminal retains the BTB domain of the *ZBTB16* gene and 2–3 zinc finger protein domains, while the C-terminal *ABL1* gene retains the kinase functional domain, resulting chimeric protein has tyrosine kinase activity. At present, Functional studies have demonstrated that the tyrosine kinase activity of *ZBTB16::ABL1* is approximately four-fold higher than that of wild-type *ABL1*, whereas *BCR::ABL1* exhibits approximately five-fold higher activity, indicating that the kinase activities of these two fusions are broadly comparable (4). Importantly, both *in vitro* and *in vivo* models have confirmed that *ZBTB16::ABL1* is sensitive to imatinib and dasatinib (4). However, clinical data on TKI therapy in *ZBTB16::ABL1*-positive T-ALL remain extremely limited, with only one prior case reported to date (16). In this case study, although combined TKI and chemotherapy induced a transient remission, the patient relapsed during consolidation therapy with CNS involvement. Whether this relapse reflects a biological propensity of *ZBTB16::ABL1* for extramedullary involvement, inadequate CNS penetration of dasatinib, or is merely coincidental remains unclear and cannot be concluded from a single case. The occurrence of CNS relapse raises the question of whether adequate CNS penetration of dasatinib may be a concern, as has been suggested in Ph+ ALL (20). However, whether higher-dose or next-generation TKIs would improve CNS outcomes in *ZBTB16::ABL1*-positive T-ALL remains speculative and requires prospective evaluation.

## Conclusion

T-cell Acute Lymphoblastic Leukemia (T-ALL) is a disease caused by the combined effect of multiple molecular abnormalities. Among them, the *ABL1* fusion gene confers proliferation and survival advantages to leukemic cells. In this report, we describe the clinical course of a pediatric T-ALL patient harboring the *ZBTB16::ABL1* fusion who received dasatinib-containing therapy, and we summarize the currently reported ABL1-rearranged T-ALL cases in the literature. Given the high prognostic heterogeneity of this specific subtype, definitive conclusions regarding its biological behavior, prognostic significance, or optimal treatment strategy cannot be drawn from a single case. Accumulation of additional cases and, where feasible, functional and pharmacokinetic studies are urgently needed to better elucidate the molecular mechanisms underlying this fusion and to guide evidence-based therapeutic decisions for this rare molecular subset.

## Data Availability

The raw data supporting the conclusions of this article will be made available by the authors, without undue reservation.
