# Supplementary material for: Case Report: Clinical response to TKI therapy in T-cell acute lymphoblastic leukemia with rare ABL1 rearrangement
Source: Front Oncol. 2026 Sep 10;16:1928646. doi: 10.3389/fonc.2026.1928646 (PMC13600990; doi:10.3389/fonc.2026.1928646)
Supplement: Supplementary file 1 [file DataSheet1.pdf]

Supplementary material

Article title: Case Report: Clinical Response to TKI Therapy in T cell Acute Lymphoblastic Leukemia with Rare ABL1 Rearrangement

Risk stratification, and treatment for high-risk acute lymphoblastic leukemia according to the SCCLG-ALL 2016 protocol:

1. Risk stratification

Low-risk patients were those who aged ≥1 and < 10 years old with initial WBC < 50 $\times 10^9$/L, prednisone-good response defined as blast < 1.0 $\times 10^9$/L in peripheral blood on day 8 of prednisone treatment, < 5% blasts in bone marrow on day 15 induction, and complete remission after induction therapy with VDLDex.

Intermediate-risk patients were those who aged <1 or ≥ 10 years old, with initial WBC ≥ 50 $\times 10^9$/L, prednisone-good response, T-ALL, initial central nervous system leukemia / testicular leukemia, E2A-PBX1 / BCR-ABL positive ALL, or ≥ 5% and < 25% blasts in bone marrow on day 15 induction, and who achieved complete remission after induction therapy with VDLDex.

The criteria of high-risk were: prednisone-poor response (blast ≥ 1.0 $\times 10^9$/L in peripheral blood on day 8 of prednisone treatment), MLLr positive, blast cells in bone marrow ≥ 25% on day 15 induction, and/or non-remission after induction therapy with VDLDex.

2. Treatment protocol for ALL-HR

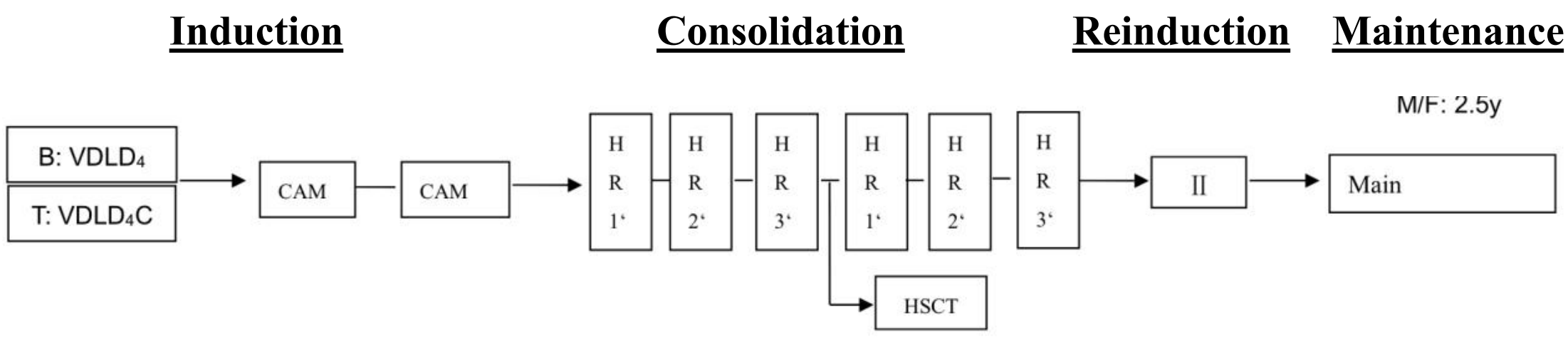

Protocol IA
Induction Therapy: IR/HR -ALL

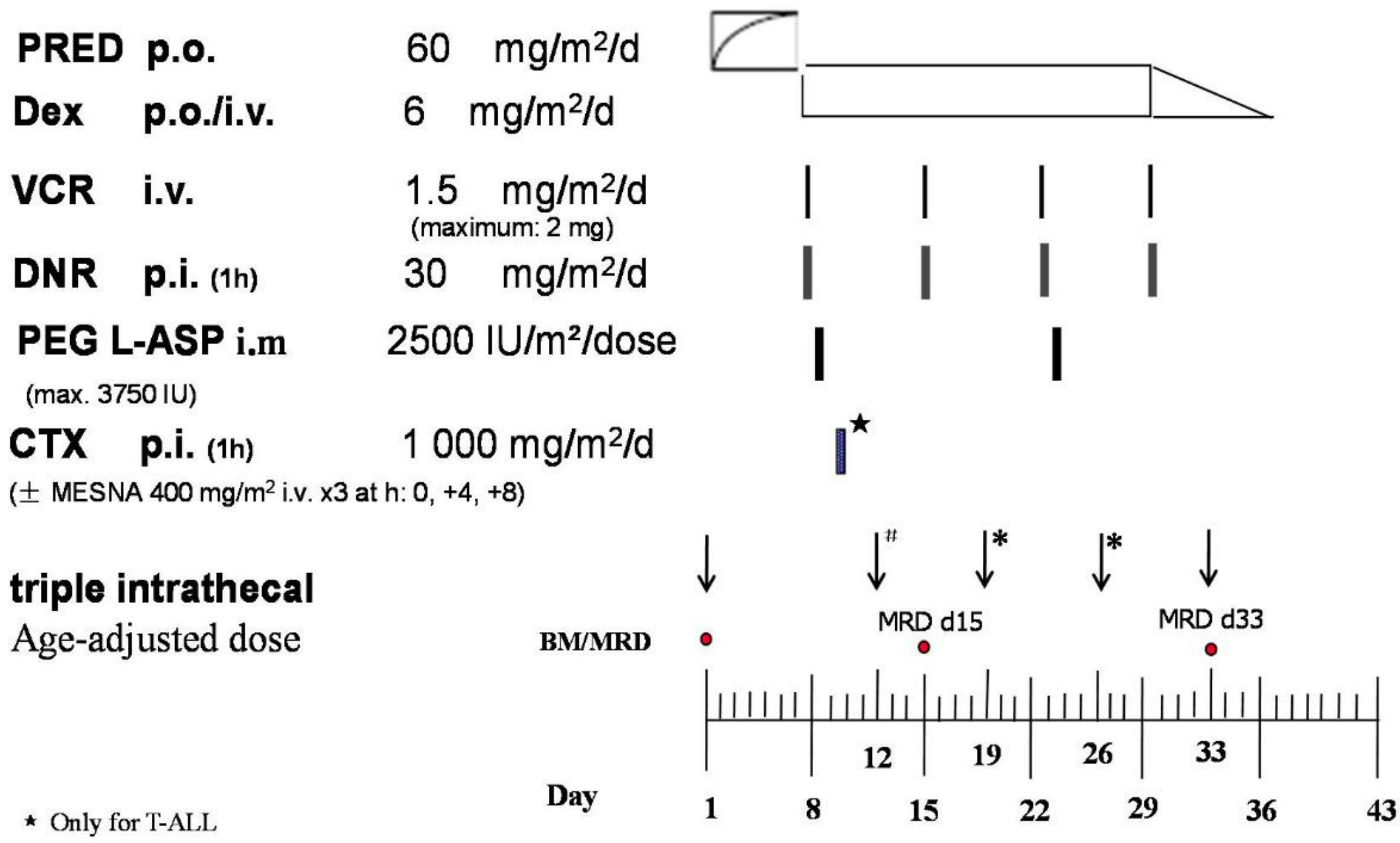

★ Only for T-ALL

# if CNS1 for practical reasons would prefer to administer IT on d15

* If CNS2, or traumatic LP: additional IT on d19 and 26

Protocol IB
Induction Therapy: HR - ALL

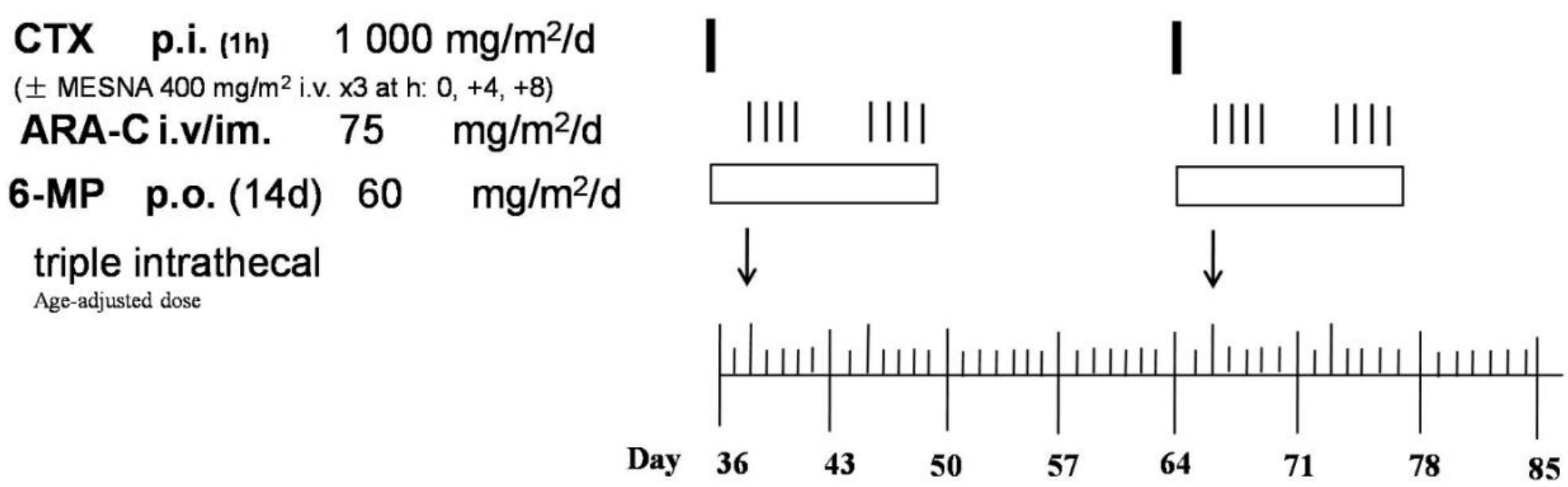

Block HR-1'

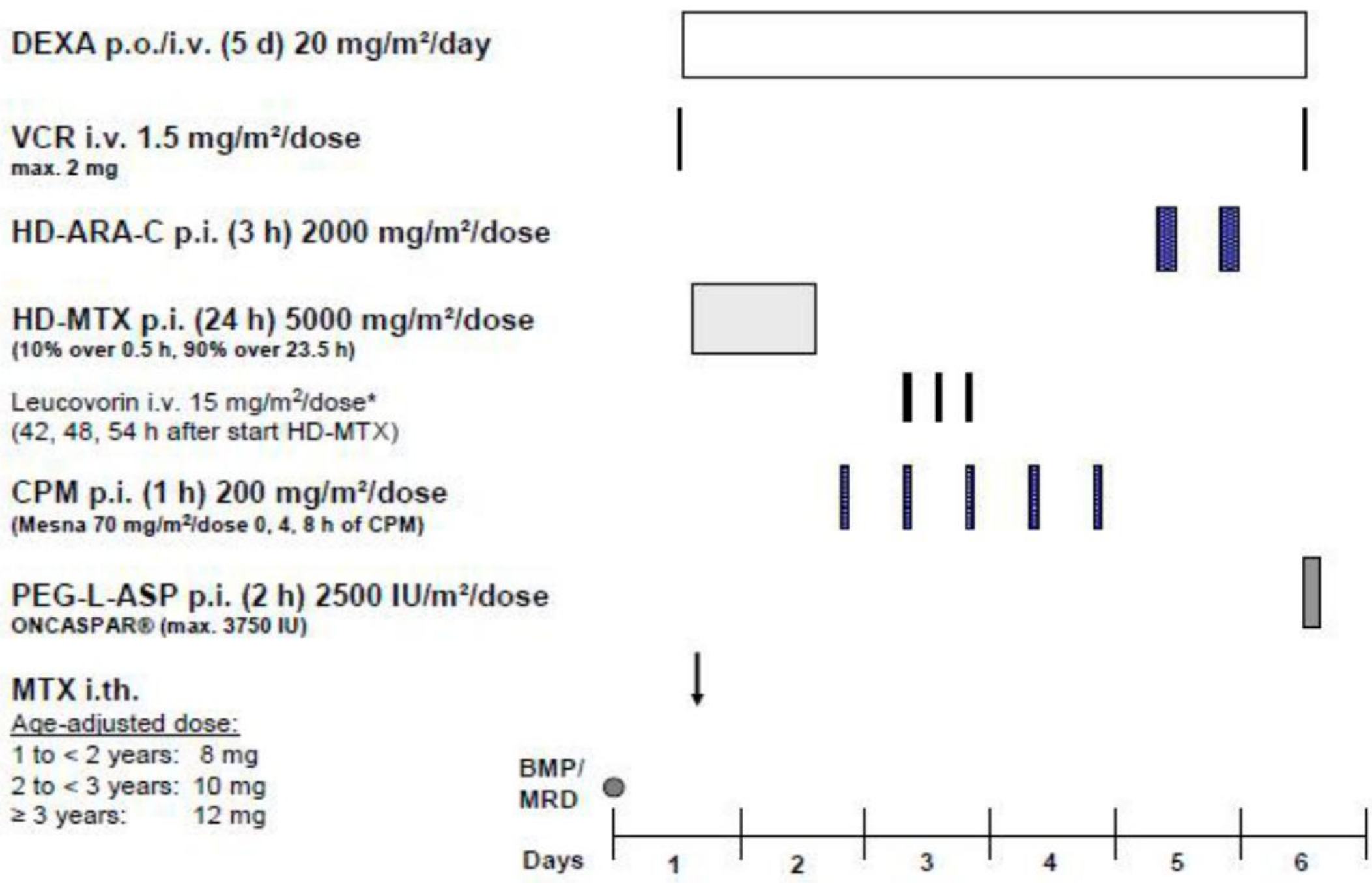

* Leucovorin i.v. 7.5 mg/m²/dose (42, 48, 54 h after start HD-MTX) if levo-product is used

Block HR-2'

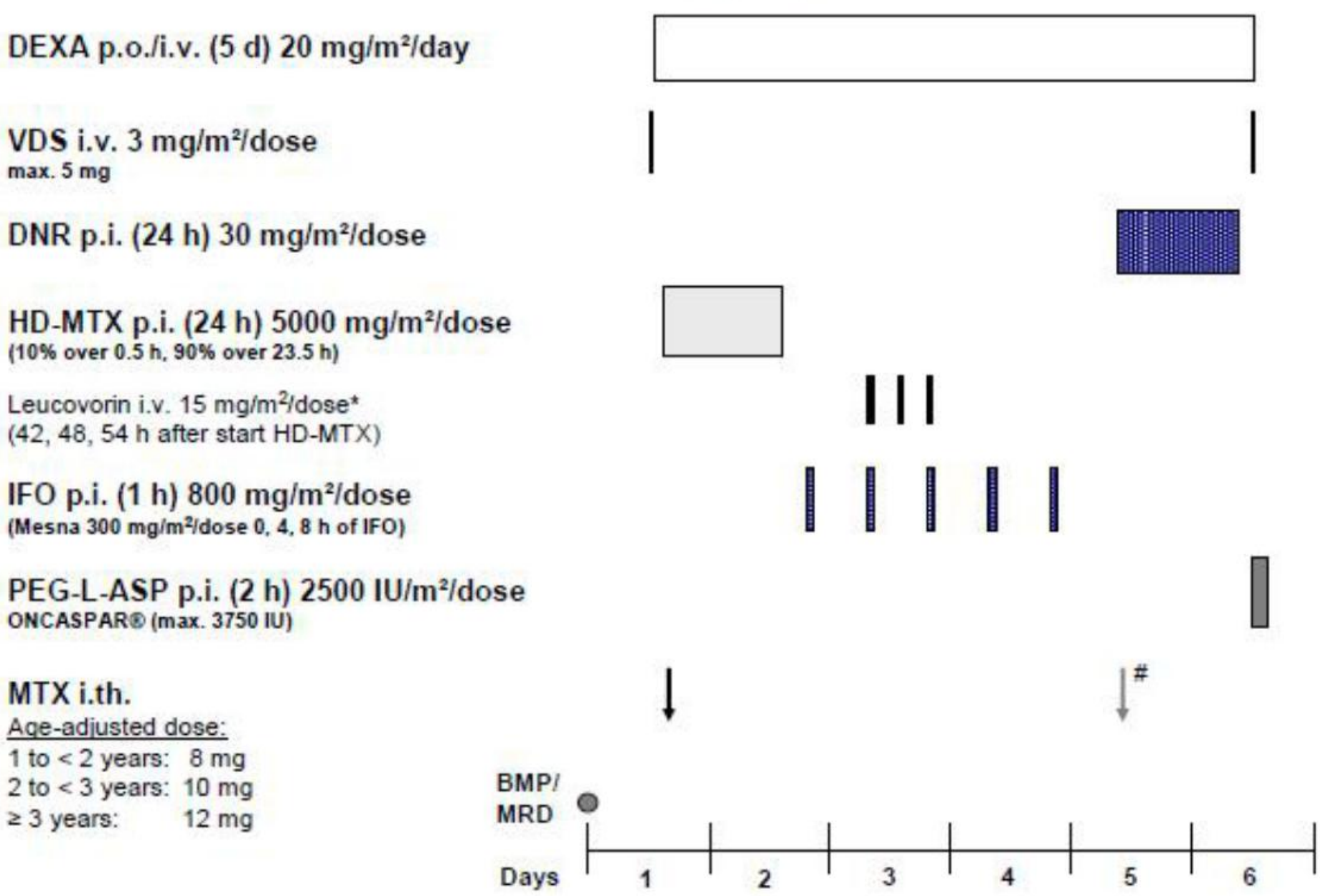

#additional i.th. MTX on day 5 if CNS-positive (CNS 3)

* Leucovorin i.v. 7.5 mg/m²/dose (42, 48, 54 h after start HD-MTX) if levo-product is used

Block HR-3'

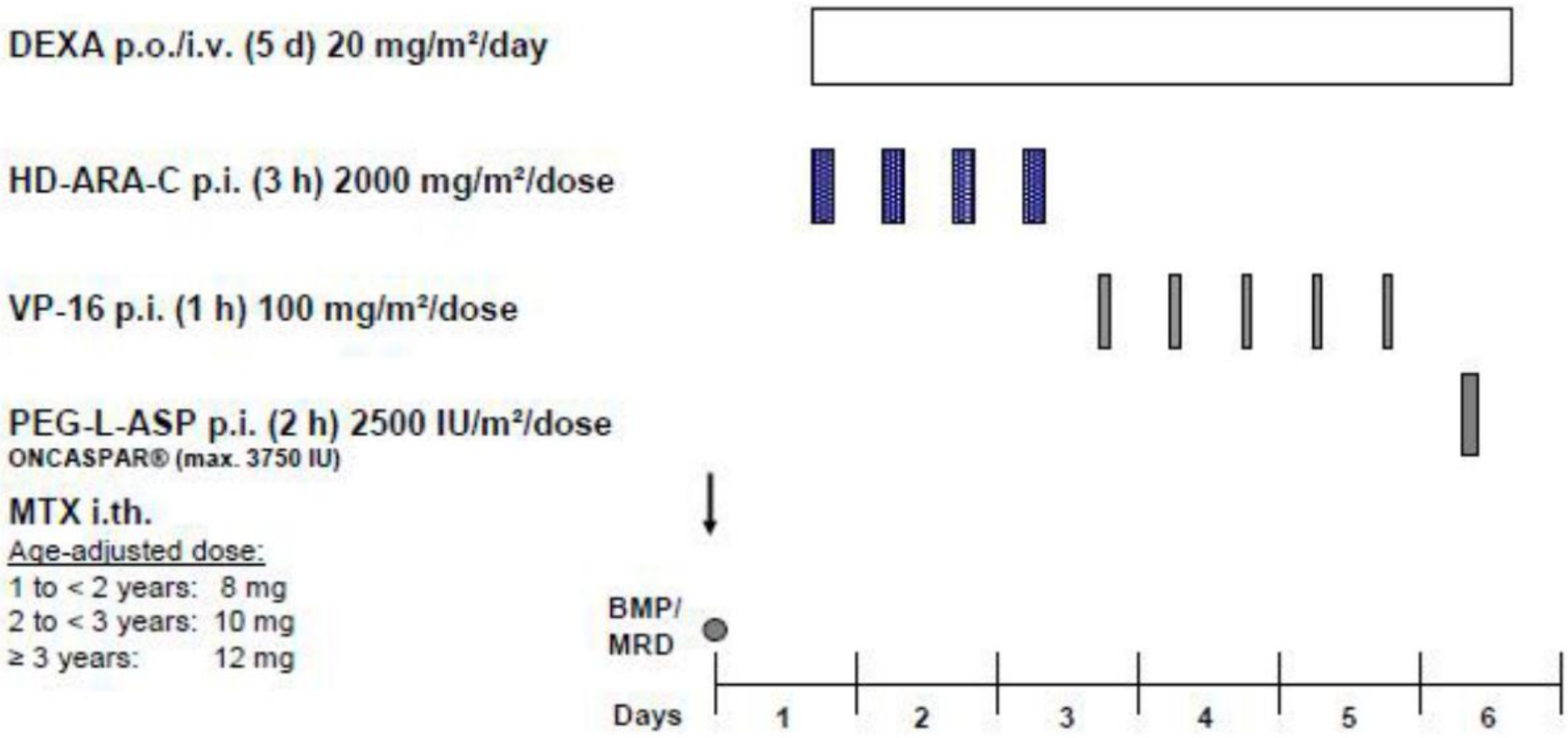

Protocol II
Reinduction Therapy: HR-ALL

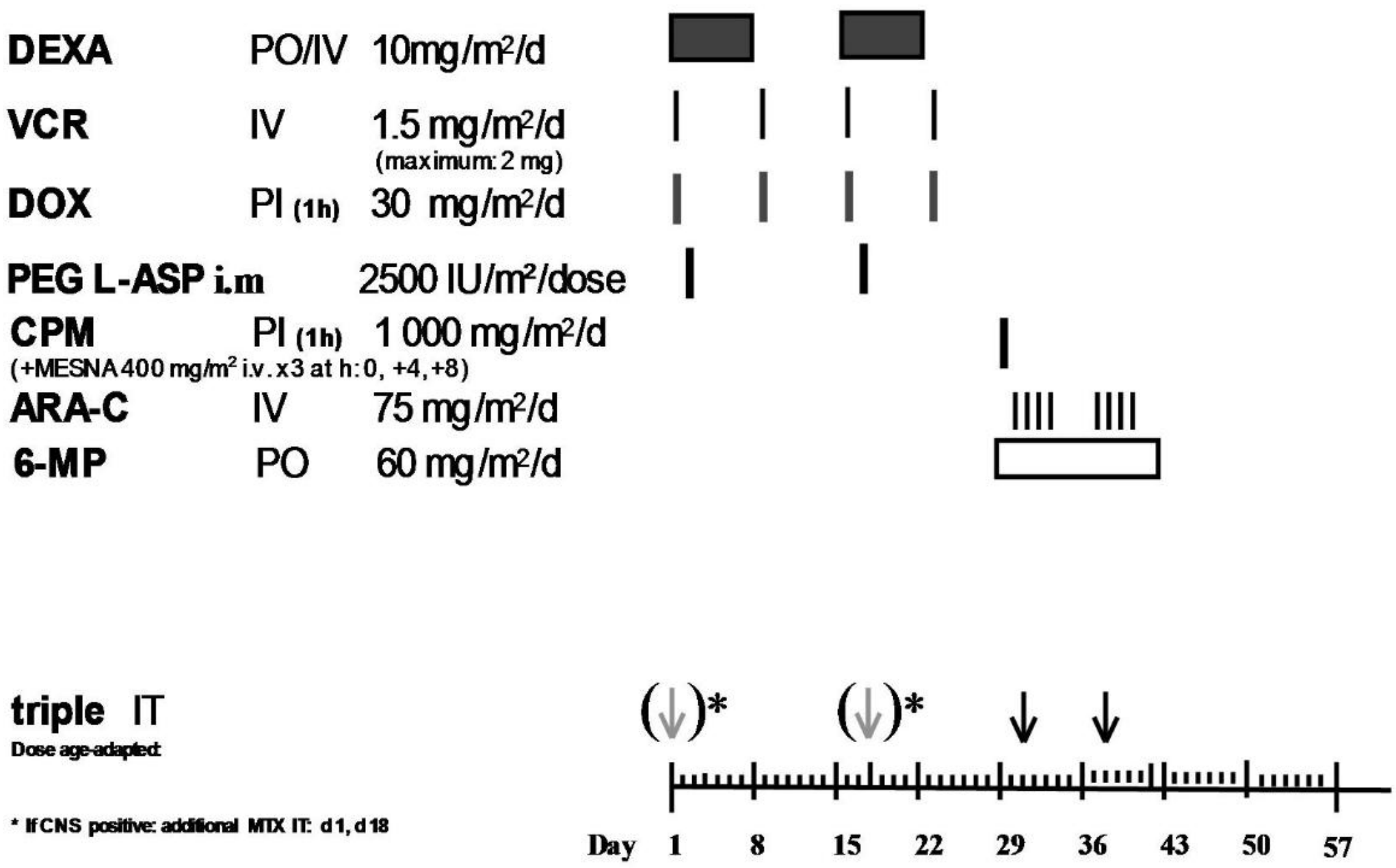

3. Intrathecal injection(IT) protocol

Age（M）	MTX(mg)	Ara-C(mg)	Dex(mg)
<12	6	12	2
~23	8	15	2
~35	10	25	5
⩾36	12	30	5
